# Short-term vagal nerve stimulation improves left ventricular function following chronic heart failure in rats

**DOI:** 10.3892/mmr.2015.3597

**Published:** 2015-04-07

**Authors:** YAN LI, YAN-HUA XUAN, SHUANG-SHUANG LIU, JING DONG, JIA-YING LUO, ZHI-JUN SUN

**Affiliations:** 1 Department of Geriatrics and Shengjing Hospital of China Medical University, Shenyang, Liaoning 110004, P.R. China; 2 Department of Cardiology Medicine, Shengjing Hospital of China Medical University, Shenyang, Liaoning 110004, P.R. China

**Keywords:** electrical stimulation, heart failure, myocardial infarction, remodeling vagus nerve

## Abstract

Increasing numbers of animal and clinical investigations have demonstrated the effectiveness of long-term electrical vagal nerve stimulation (VNS) on chronic heart failure (CHF). The present study investigated the effects of short-term VNS on the hemodynamics of cardiac remodeling and cardiac excitation-contraction coupling (ECP) in an animal model of CHF following a large myocardial infarction. At 3 weeks subsequent to ligation of the left coronary artery, the surviving rats were randomized into vagal and sham-stimulated groups. The right vagal nerve of the CHF rats was stimulated for 72 h. The vagal nerve was stimulated with rectangular pulses of 40 ms duration at 1 Hz, 5 V. The treated rats, compared with the untreated rats, had significantly higher left ventricular ejection fraction (54.86±9.73, vs. 45.60±5.51%; P=0.025) and left ventricular fractional shortening (25.31±6.30, vs. 15.42±8.49%; P=0.013), and lower levels of brain natriuretic peptide (10.07±2.63, vs. 19.95±5.22 ng/ml; P=0.001). The improvement in cardiac pumping function was accompanied by a decrease in left ventricular end diastolic volume (1.11±0.50, vs. 1.54±0.57 cm^3^; P=0.032) and left ventricular end systolic volume (0.50±0.28, vs. 0.87±0.36 cm^3^; P=0.007). Furthermore, the expression levels of ryanodine receptor type 2 (RyR2) and sarcoplasmic reticulum calcium adenosine triphosphatase (SERCA2) were significantly higher in the treated rats compared with the untreated rats (P=0.011 and P=0.001 for RyR2 and SERCA2, respectively). Therefore, VNS was beneficial to the CHF rats through the prevention of cardiac remodeling and improvement of cardiac ECP.

## Introduction

Heart failure is an important public health concern, with a high mortality rate and poor treatment response. Previous studies have revealed that there is a close association between the pathogenesis of chronic heart failure (CHF) and imbalance of the autonomic nervous system, which is manifested by increased sympathetic activity and reduced parasympathetic activity (1,2).

Adjusting the imbalance of the autonomic nervous state has been confirmed to relieve the symptoms and improve the prognosis of CHF (3–6). In addition, accumulating animal and clinical investigations have demonstrated the effectiveness of electrical vagal nerve stimulation (VNS) on CHF (7–15). However, the majority of studies have focused on the effect of long-term VNS, and the effect of short-term VNS on CHF remain to be elucidated. The present study aimed to evaluate the effect of short-term VNS on CHF.

The present study investigated the effects of short-term VNS on the hemodynamics, cardiac remodeling and cardiac excitation-contraction coupling in an animal model of CHF following large myocardial infarction. The aim of the present study was to investigate whether short-term VNS was a benefit to CHF and whether short-term VNS during CHF prevented cardiac remodeling and improved the cardiac excitation-contraction coupling.

## Materials and methods

### Animals

Male Wistar rats (7–8 weeks old; weighing 250–300 g) were obtained from the Animal Center of the Chinese Academy of Medical Sciences (Beijing, China) for use as a model of CHF in the present study. The animals were housed in stainless steel wire-mesh cages (5 rats per cage) under standard laboratory conditions (25°C, relative humidity 60%, 12 hours dark/light periods) and were allowed free access to water and food. The investigations conformed to the Guide for the Care and Use of Laboratory Animals, published by the National Institutes of Health (NIH Publication no. 85–23; revised 1985). The present study was approved by the Animal Research Ethics Committee of Shengjing hospital of China Medical University (Shenyang, China). The rats were assigned into three different treatment groups: Rats with CHF, treated with sham stimulation (CHF-SS group; n=9), rats with CHF, treated with VNS (CHF-VNS group; n=9) and sham-operated rats, treated with sham stimulation (SO-SS group; n=5).

### Rat model of CHF

Acute myocardial infarction was induced in the rats by left anterior descending artery ligation, as previously described (16). Briefly, the rats were anesthetized via intraperitoneal injection of 10% chloral hydrate (3 ml⁄kg). The rats were then tracheotomied, intubated and mechanically ventilated, with arterial pH, PO_2_ and PCO_2_ maintained within the physiological range by supplying O_2_ and altering the respiratory rate using an aHX-300S Animal Respirator (Chengdu Tme Technology Co, Ltd., Chengdu, China). A thoracotomy was performed through the fourth intercostal space, the heart was exposed and an electrocardiogram was monitored on an BL-420S Data Acquisition & Analysis System (Chengdu Tme Technology Co, Ltd., China). A prolene suture (Ethicon, Inc., Somerville, NJ, USA) was placed around the left coronary artery, close to its origin, and the ends were tied firmly in the CHF-SS and CHF-VNS groups and loosely in the SO-SS group. Acute myocardial infarction was deemed successful on the basis of regional cyanosis of the myocardial surface distal to the suture, accompanied by elevation of the ST segment on the electrocardiogram. At 3 weeks-post myocardial infarction, echocardiography was performed on the surviving rats. The rats, which exhibited left ventricular wall infarction, with an infarction area >40% of the left ventricular wall area were enrolled for the subsequent investigations. The infarcted rats were randomly allocated into the CHF-VNS group (n=9) or CHF-SS group (n=9). Heart rate, mean blood pressure, weight, and echocardiography were recorded, and blood samples were obtained at baseline and after 72 h in the rats of the two groups. The present study was approved by the ethics committee of Shengjing Hospital of China Medical University (Shenyang, China).

### VNS

VNS was performed 3 weeks after the ligation of the left anterior descending artery. Under general anesthesia via intraperitoneal injection of 10% chloral hydrate (3 ml⁄kg; Experimental Animal Center of Shengjing Hospital of China Medical University, Shenyang, China), a pair of Teflon-coated stainless steel wires (UL1330; Triumph Cable Co, Ltd, Dongguan, China) were looped around the right vagal nerve in the neck for electrical stimulation. This was connected to the output terminals of the stimulator (BL-420S Data Acquisition & Analysis System; Chengdu Tme Technology Co, Ltd.), which provides stimulation over a range of frequencies (0.1–100 Hz), strengths (1–10 V) and pulse widths (0.001–10 sec.). In the present study, the vagal nerve was stimulated with rectangular pulses of 40 ms duration at 1 Hz, 5 V. To prevent drying and to provide insulation, the stimulation electrodes and the vagus nerve were immersed in a mixture of white petrolatum (Vaseline) and paraffin.

### Echocardiographic and hemodynamic measurements

Echocardiography was performed 3 weeks after surgery. A transthoracic 2D M-mode echocardiogram was obtained using a Philips iE33 (Philips Electronics, Amsterdam, The Netherlands), equipped with a transducer S12–4. The rats were anesthetized with 3 ml/kg 10% chloral hydrate, and echocardiography was performed. The chest each mouse was shaved, and the rats were placed in a supine position. Images were captured by placing the transducer against the chest. The left ventricular (LV) area was imaged in the short-axis view at the mid-papillary muscle, and M-mode measurements of the LV end-diastolic diameter and LV end-systolic diameter were recorded. The LV ejection fraction (LVEF) and LV fractional shortening (LVFS) were measured using the Teichholtz method.

### Blood sampling and tissue preparation

Blood samples (2 ml whole blood) were collected from each rat rapidly via the arterial catheter into a syringe (WeiGao group medical polymer Co., Ltd., Shundong, China) at the baseline (0 h), prior to treatment, and again following 72 h of treatment. The rats were sacrificed by spinne dislocation at the end of the experiments. For each rat, the entire heart was rapidly excised and washed with cold phosphate buffer (Experimental Center of Shengjing Hospital of China Medical University, Shenyang, China, containing 137 mmol/l NaCl, 2.7 mmol/l KCl, 10 mmol/l Na_2_HPO_4_, 2 mmol/l KH_2_PO_4_ (pH 7.4). The heart was then frozen at −80°C, prior to its use in the subsequent reverse transcription-quantitative polymerase chain reaction (RT-qPCR) and western blot analyses.

### Detection of brain natriuretic peptide (BNP)

The blood samples were collected into serum separator tubes, and serum was obtained by centrifugation at 4,500 g for 15 min. The serum BNP was measured using a commercially available human/mouse/rat BNP enzyme immunoassay kit (RayBiotech, Norcross, GA, USA), according to the manufacturer’s instructions. All samples and standards were measured in triplicate.

### RT-qPCR

Total cellular RNA was extracted fro the tissues using the RNeasy Mini kit from Qiagen (Hilden, Germany). RT was performed with 1 *µ*g RNA using a PrimeScript RT Reagent kit (Takara Bio, Inc., Dalian, China), according to the manufacturer’s instructions. A total of 2 *µ*l of 50 ng/*µ*l cDNA (produced from reverse transcription) was used for qPCR. qPCR was then performed using SYBR Premix Ex Taq II (Takara Bio, Inc.) on a 7500 Real-time PCR system (Applied Biosystems Life Technologies, Foster City, CA, USA) as follows: 50°C for 2 min, 95°C for 10 min, 40 cycles of 95°C for 15 sec, and 60°C for 60 sec. DNA hybridization sequencing was performed for confirmation of the amplification specificity (17). β-actin was used as the internal reference gene. The relative levels of gene expression were represented as ΔCt = Ct gene – Ct reference, and the fold changes in gene expression levels were calculated using the 2^−ΔΔCt^ method (18). The experiments were performed in triplicate. The sequences of the primer pairs used are as follows: β-actin, forward 5′-ATAGCACAGCCTGGATAGCAACGTAC-3′, and reverse 5′-CACCTTCTACAATGAGCTGCGTGTG-3′; ryanodine receptor 2 (RyR2), forward 5′-CCCACCTCCTTGACATCG-3′ and reverse 5′-CAGCCAACAAGCCAACAG-3′; sarcoplasmic reticulum calcium adenosine triphosphatase (SERCA2), forward 5′-TGATGACATGATGACGTGCTA-3′ and reverse 5′-TCAAAGACGATGCGATACA-3′, all synthesized by Double Gene Technology Co., Ltd., Beijing, China).

### Western blot analysis

The total proteins from the tissues were extracted in 10X cell lysis buffer (Pierce Biotechnology, Inc., Rockford, IL, USA) and quantified using the Bradford method. Samples of 50 g of protein were separated by SDS-PAGE (12%; Experimental Center of Shengjing Hospital of China Medical University, Shenyang, China). The proteins were transferred onto polyvinylidene fluoride membranes (Millipore, Billerica, MA, USA), blocked with 15% non-fat milk (Wanda Emulsion Co., Ltd., Heilongjiang, China) and incubated overnight at 4°C with antibodies against SERCA2 (mouse polyclonal; 1:500; cat. no. ab2861; Abcam, Cambridge, MA, USA), RyR2 (rabbit polyclonal; 1:1,000; cat. no. AAR-002; Cell signaling Technology, Inc., Boston, MA, USA) and GAPDH (rabbit poly-clonal; 1:2,000; cat. no. ARP58578_P050; American Research Products, Inc., Belmont, MA, USA). Following incubation with peroxidase-coupled mouse immunoglobulin G (1:3,000, Santa Cruz Biotechnology, Inc., Dallas, TX, USA) at 37°C for 2 h, the bound proteins were visualized using enhanced chemiluminescence (Pierce Biotechnology, Inc.), and detected using a Bioimaging system (UVP Inc., Upland, CA, USA).

### Histological analysis

The middle ring slice of the left ventricle was embedded in paraffin using an TB-718D biological tissue automatic embedding machine (Hubei Taiwei Technology Industrial Co., Ltd., Hubei, China), sectioned at a thickness of 5 *µ*m and stained with hematoxylin and eosin (H&E; Huayueyang Biological Technology Co., Ltd., Beijing, China), following which they were observed under light microscopy (Eclipse E800 light microscope; Nikon, Tokyo; Japan).

### Statistical analysis

All statistical analyses were performed using SPSS 13.0 software (SPSS, Inc., Chicago, IL, USA). Data are expressed as the mean ± standard deviation. Statistical analyses were performed using Student’s t-test for comparison between two groups GraphPad Prism). P<0.05 was considered to indicate a statistically significant difference.

## Results

### Characteristics of the rat models

Prior to VNS, the baseline characteristics of the rats in the CHF-SS and CHF-VNS groups were compared. As shown in Table I, no statistical differences were observed in heart rate (HR), blood pressure (BP), weight, LV end-diastolic volume (LVEDV), LV endsystolic volume (LVESV), LVFS or LVEF between the two groups (P>0.05).

### Effect of short-term VNS on hemodynamics

**Figure 1:**
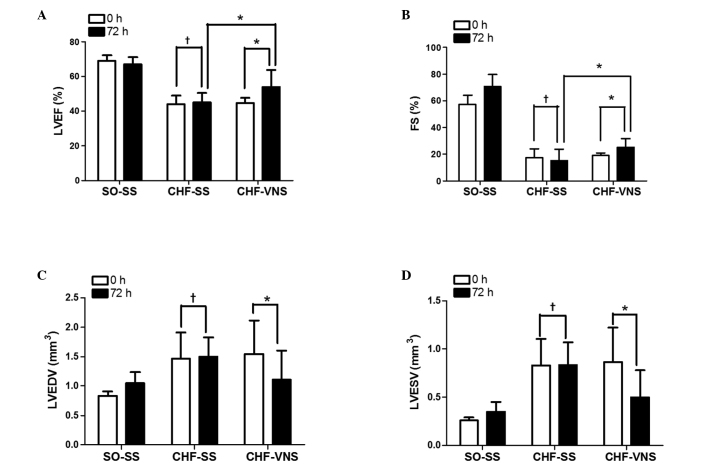
Levels of (A) LVEF, (B) LVFS, (C) LVEDV and (D) LVESV prior to and following treatment in the SO-SS (n=5), CHF-SS (n=9) and CHF-VNS (n=9) treatment groups. Assessment was performed prior to, and treatment for 72 h. Data are presented as the mean ± standard deviation. ^†^P>0.05 and ^*^P<0.05, compared with 0 h. SO, sham-operated; CHF, chronic heart failure; SS, sham stimulation; VNS, vagal nerve stimulation; LVEF, left ventricular ejection fraction; LVFS, left ventricular fractional shortening; LVEDV, left ventricular end-diastolic volume; LVESV, left ventricular end-systolic volume.

Prior to VNS, the LVEF was markedly lower in the CHF rats compared with the SO-SS rats, whereas no significant difference were observed between the CHF-VNS and CHF-SS groups (Table I). As shown in Fig. 1A, 72 h following VNS, the LVEF values were significantly increased (P=0.007) in the CHF-VNS group, while no significant changes were observed in the CHF-SS group (P=0.464). The LVEF increased significantly in the CHF-VNS group, compared with the CHF group, following VNS (54.86±9.73, vs. 45.60±5.51%, respectively; P=0.025). Accordingly, 72 h after VNS, the LVFS value was increased significantly in the CHF-VNS group compared with the CHF-SS group (25.31±6.30, vs. 15.42±8.49%, respectively; P=0.013; Fig. 1B).

In addition, short-term VNS reduced the LVEDV and the LVESV in the CHF-VNS rats (1.11±0.50, vs. 1.54±0.57 cm^3^; P=0.032 and 0.50±0.28, vs. 0.87±0.36 cm^3^, respectively; P= 0.007), as shown in Fig. 1C and D. However, no significant changes in LVEDV or LVESV were observed in the CHF-SS rats following VNS (1.50 ± 0.33, vs.1.47±0.44 cm^3^; P=0.87 and 0.83±0.23, vs. 0.83±0.27 cm^3^, respectively; P=0.984), as shown in Fig. 1C and D).

### Effect of short-term VNS on the expression of BNP

**Figure 2:**
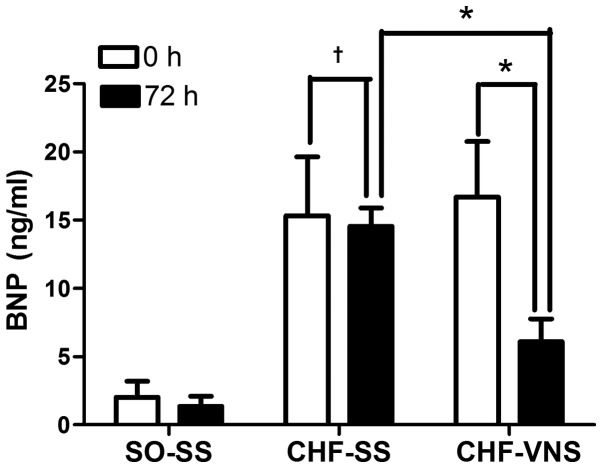
Serum levels of BNP prior to and following treatment in the SO-SS (n=5), CHF-SS (n=9) and CHF-VNS (n=9) treatment groups. Assessment was performed prior to (0 h) and following treatment for 72 h. Data are presented as the mean ± standard deviation. ^†^P>0.05 and ^*^P<0.05, compared with 0 h. BNP. brain natriuretic peptide; SO, sham-operated; CHF, chronic heart failure; SS, sham stimulation; VNS, vagal nerve stimulation.

The effect of short-term VNS on the serum levels of BNP were evaluated 72 h after VNS. Prior to stimulation, the serum level of BNP was significantly higher in the CHF rats compared with the SO-SS rats, and no significant differences were observed between the CHF-SS and CHF-VNS groups (Table I). As shown in Fig. 2, following short-term VNS, serum levels of BNP decreased significantly in the rats of the CHF-VNS group (10.07±2.63, vs. 23.80±5.82 ng/ml, respectively; P=0.001), while the level of BNP did not alter significantly in the CHF-SS rats (19.95±5.22, vs. 23.56± 5.71 ng/ml, respectively; P>0.05). VNS decreased the serum levels of BNP significantly in the CHF-VNS rats compared with the CHF-SS rats, (10.07±2.63, vs. 19.95±5.22 ng/ml, respectively; P<0.001).

### Effect of short-term VNS on cardiac excitation-contraction coupling (ECP)

**Figure 3:**
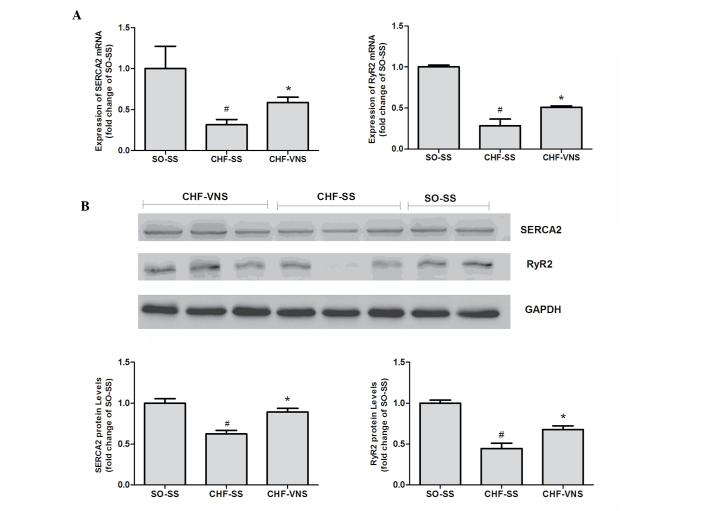
Myocardial mRNA and protein expession levels of RYR2 and SERCA2 in the SO-SS (n=5), CHF-SS (n=9) and CHF-VNS (n=9) treatment groups. (A) mRNA expression levels of RYR2 and SERCA. (B) Representative western blot of RYR2 and SERCA2, and corresponding GAPDH bands. The band intensities were normalized to the SO-SS values shown. Assessment was made following treatment for 72 h. Data are presented as the mean ± standard deviation. ^*^P<0.05, compared with CHF-SS; ^#^P<0.05, compared with SO-SS.

RyR2 and SERCA2 are core components of cardiac ECP. To examine the effect of short-term VNS on the expression levels of RyR2 and SERCA2, qPCR and western blot analyses were performed. As shown in Fig. 3, the mRNA expression levels of RyR2 and SERCA2 were significantly lower in the CHF-SS group compared with the SO-SS group (P<0.000), however, the levels of RyR2 and SERCA2 were increased in the CHF-VNS group compared with the CHF-SS group (P=0.017 and P=0.008, respectively). Consistent with the qPCR results, the protein expression levels of RyR2 and SERCA2 were significantly lower in the CHF-SS group compared with the SO-SS group (P=0.001 and P<0.000, respectively), and were higher in the CHF-VNS group compared with the CHF-SS group (P=0.011 and P=0.001, respectively).

### Effect of short-term VNS on histological changes in the CHF rat model

**Figure 4:**
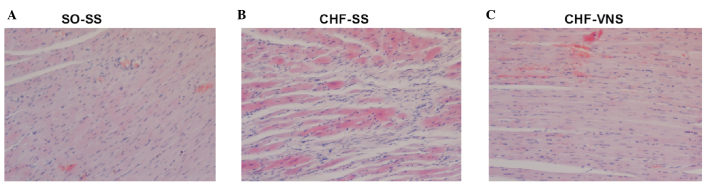
Photomicrographs (magnification, x100) of hematoxylin and eosin staining of infarcted regions of left ventricle cross-sections. (A) SO-SS treatment for 72 h caused no obvious pathological change, the myocardial striations, intercalated discs and cell nuclei were clearly stained, the cardiomyocytes exhibited regular shape and arrangement with a clear edge, and the cytoplasm and nuclei were stained evenly. In addition, the cardiac myofibers were tightly connected and the interstitial fibroblasts displayed no morphological abnormality (n=5). (B) CHF-SS treatment for 72 h caused cardiomyocytes to exhibit an irregular morphology and arrangement. The cytoplasm was unevenly stained with irregular granules, the nuclei were condensed and darkly stained. In addition, myofibers swelling, interstitial edema and red blood cell leakage were also observed (n=9). (C) CHF-VNS treatment resulted in cardiomyocytes being notably disorganised, the myocardial cells were arranged relatively neatly, the intercalated discs and nucleus appeared more clear (n=9). SO, sham-operation; SS, sham stimulation; CHF, chronic heart failure; VNS, vagal nerve stimulation.

H&E staining was performed on sections of ventricular tissue in the rats models to evaluate the effect of VNS on histology (Fig. 4). In the SO-SS rats, no obvious pathological changes were observed; the myocardial striations, intercalated discs and cell nuclei were clearly stained; the cardiomyocytes exhibited regular shape and arrangement with clear edges, and the cytoplasms and nuclei stained evenly. In addition, the cardiac myofibers were tightly connected and the interstitial fibroblasts exhibited no morphological abnormality. However, the cardiomyocytes in the CHF-SS rats exhibited irregular morphology and arrangement. The cytoplasms stained unevenly with irregular granules, the nuclei were condensed and stained darkly, and myofiber swelling, interstitial edema and red blood cell leakage were observed. By contrast, following short-term VNS, the disorganization of cardiomyocytes was reduced in the CHF-VNS group, the myocardial cells appeared relatively neatly arranged and the appearances of the intercalated discs and nuclei were clearer.

### Assessment of safety

**Figure 5:**
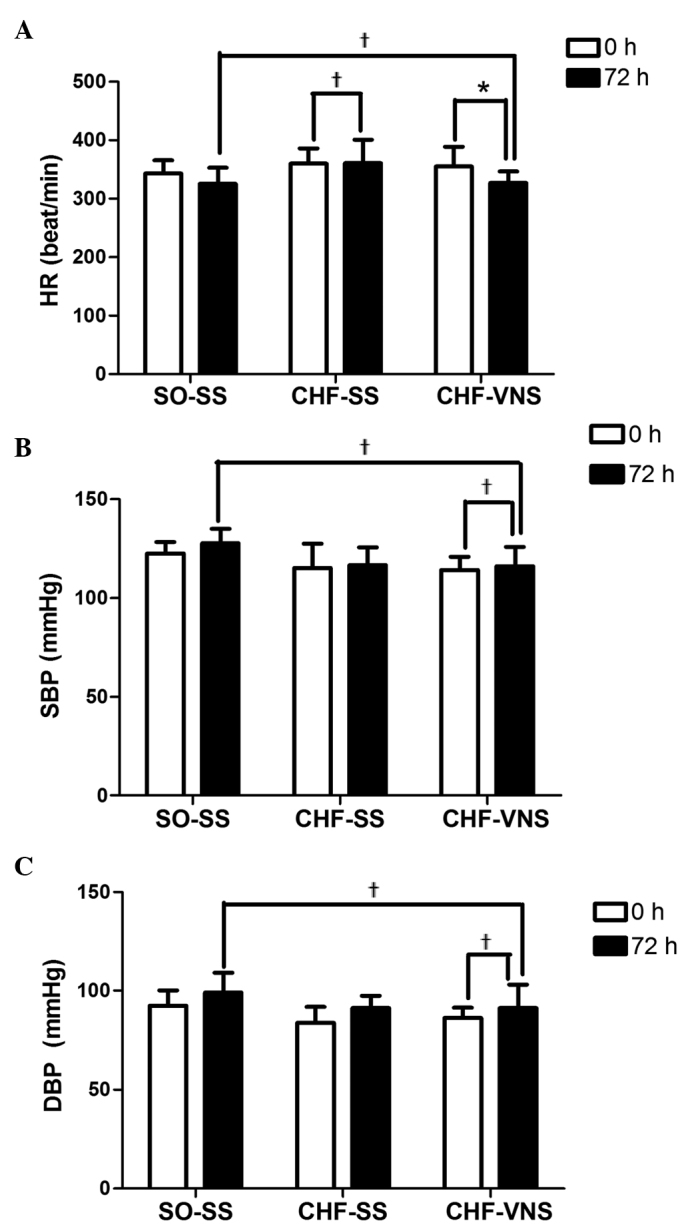
Effect of VNS on HR and BP. The (A) HR and (B and C) BP were assessed in the SO-SS (n=5), CHF-SS (n=5) and CHF-VNS treatment groups. Assessment was made prior to (0 h) and following treatment for 72 h. Data are presented as the mean ± standard deviation. ^†^P>0.05 and ^*^P<0.05, compared with 0 h. HR, heart rate; SBP, systolic blood pressure; DBP, diastolic blood pressure. SO, sham-operated; SS, sham stimulation; CHF, chronic heart failure; VNS, vagal nerve stimulation.

To assess the safety of short-term VNS in the treatment of CHF, the present study examine the effect of VNS on HR and BP. As shown in Fig. 5A, the HR of the CHF-VNS rats decreased significantly 72 h after VNS (326.89±19.73, vs. 354.67±33.95 bpm; P=0.028), while the HR in the untreated CHF rats remain unchanged (360.67±39.88, vs. 360.00±25.89 bpm; P=0.918). Notably, no significant differences were observed between the HR reduction in the CHF-VNS rats compared with the SO-SS rats (326.89±19.73, vs. 325.60±26.91 bpm; P=0.919).

As shown in Fig. 5B and C, no significant differences were observed in the CHF-VNS group, prior to and following VNS treatment, in the SBP (114.22±7.00, vs. 116.00±9.82 mmHg, respectively; P=0.484) or DBP (86.33±5.10, vs. 91.33±11.88 mmHg, respectively; P=0.325). Following short-term VNS, no significant differences were observed between the CHF-VNS and SO-SS group in SBP (116.00±9.82, vs. 122.20±8.70 mmHg, respectively; P=0.263) or DBP (91.33±11.88, vs. 91.00±8.06 mmHg, respectively; P=0.957).

## Discussion

The prognosis of patients with CHF remains poor, despite various therapeutic approaches being currently available. Therefore, more effective modalities of therapy are required. Accumulating evidence has revealed a close association between imbalance of the autonomic nervous system and the pathogenesis of CHF. Suppressed vagal tone and overactivated sympathetic drive accelerate cardiac remodelling and increase the risk of life-threatening tachyarrhythmia. Several animal experiments and clinical investigations have reported that electrical stimulation of the vagal nerve improves the prognosis of CHF (11,12,16–33). It has been demonstrated that vagal stimulation can slow HR (11,20–23), attenuate inflammatory reactions through the regulation of tumor necrosis factor (24–26), and decrease the apoptosis of cardiomyocytes in LV21 (34). In addition, previous studies have revealed that VNS reduces epinephrine and norepinephrine, and promotes the release of acetylcholine (28,29). Furthermore, VNS attenuates cardiac remodelling through the cholinergic anti-inflammatory pathway, and markedly improves LVF (7,27,30,31). Li *et al* (11) reported that VNS markedly improves long-term survival rates in CHF rats through the prevention of cardiac remodeling. The majority of previous studies have investigated the effect of long-term VNS, and the effect of short-term VNS on CHF remain to be elucidated. The present study aimed to evaluate the therapeutic effect of short-term VNS in CHF.

The results of the present study demonstrated that short-term VNS significantly increased LVEF and reduced serum BNP in CHF rats. Compared with untreated CHF rats, the LVEF increased markedly and the serum BNP decreased significantly in CHF-VNS rats 72 h after VNS.

ECC is the basic mechanism of cardiac pump function, and RyR2 and SERCA2 are two core proteins in ECC (35–38). The activation of RyR2 channels ultimately leads to increased calcium release from the SR, which, in turn, initiates muscle contraction. The activation of SERCA2 immediately pumps the majority of Ca2^+^ back to the SR, which diastoles the myocardial fibers. In the present study, the mRNA and protein expression levels of RyR2 and SERCA2 were investigated. Prior to treatment, the expression levels of RyR2 and SERCA2 were significantly lower in the CHF group compared to those in the SO-SS group. Following VNS, the expression levels of RyR2 and SERCA2 were significantly higher in the CHF-VNS group compared with those in the CHF-SS group. These results indicated that VNS restored the expression of RyR2 and SERCA2, which enhanced ECC. This may be one important reason for which short-term VNS relieved the symptoms of CHF.

In addition, the present study found that short-term VNS reduced LVEDV and LVESE in the CHF-VNS rats. Prior to treatment, no significant differences were present in the levels of LVEDV and LVESV between the CHF-SS group and CHF-VNS group. However, 72 h after VNS, the levels of LVEDV and LVESE in the CHF-VNS group decreased significantly compared with those in the CHF-SS group, which suggested that short-term VNS not only improved the symptoms of CHF, but also attenuated LV remodeling. This indicated another possible mechanism by which short-term VNS improved the treatment of CHF.

The present study also assessed the safety of short-term VNS on CHF rats by observing the changes of HR and BP prior to and following stimulation. Prior to treatment, the HR of the CHF group were higher compared with that of the SO-SS group. At 72 h post-VNS, the HR in the CHF-VNS group decreased nearly 30 bpm, which was statistically different compared with that of CHF-SS rats, and was comparable with that of SO-SS rats. This suggested that short-term VNS decreased the HR of the CHF rats to a normal range. Accordingly, short-term VNS had no significant effect on BP. Therefore, short-term VNS was confirmed as safe for CHF treatment.

Due to their negative inotropic action and negative dromotropic action, β-blockers are routinely used in CHF, however, they are not suitable for the acute stage of severe CHF as β-blockers may lead to a decreased heart rate, decreased myocardial contractility, decreased cardiac output and a partial decrease in blood pressure resulting in severe heart failure (39). A number of studies have suggested that VNS improves long-term survival rates in CHF, however, its effect on patients with an acute stage of severe CHF, and whether short-term VNS attenuates or deteriorates symptoms, as with β-blockers in acute severe CHF, remains to be elucidated. In the present study short-term VNS was demonstrated to be effective in the acute stage of CHF, providing an alternative therapeutic option for treatment. In addition, pain and infection in certain patients may render them unsuitable for device implantation. However, stimulation electrodes are relatively simple to install and do not require device implantation (40), enabling surgery to be performed first, followed by short-term VNS for a few days. If the patients’ symptom relieve following short-term VNS and the patients can tolerate the stimulation, device implantation therapy may then be performed.

In conclusion, the present study demonstrated that short-term VNS relieved the symptoms of CHF in rats without clear side effects. These beneficial effects of short-term VNS may be attributed to the attenuation of LV remodeling and altered ECC-associated protein. These results suggested that electric stimulation may become an efficient, economical and simple method to treat CHF in the future. Further, larger-scale trials are required to examine the effects and underlying molecular mechanisms. in more depth.

## Figures and Tables

**Table I:** Baseline characteristics of the SO-SS, CHF-SS and CHF-VNS groups prior to treatment.

Characteristic	SO-SS (n=5)	CHF-SS (n=9)	CHF-VNS (n=9)
HR (bpm)	343.20±22.40	360.00±25.89	354.67±33.95^a^
SBP (mmHg)	122.60±5.68	115.22±12.35	114.22±7.00^a^
MBP (mmHg)	102.20±5.81	94.00±8.11	95.33±4.56^a^
DBP (mmHg)	92.40±7.80	83.67±8.15	86.33±5.10^a^
Weight (g)	479.80±6.65	421.67±36.02	417.11±36.52^a^
LVEDV (cm^3^)	0.83±0.07	1.47±0.44	1.54±0.57^a^
LVESV (cm^3^)	0.26±0.03	0.83±0.27	0.87±0.36^a^
LVFS (%)	57.40±6.69	17.52±6.68	19.43±1.50^a^
LVEF (%)	69.06±3.32	44.10±4.82	44.64±3.10^a^

Assessment was made prior to treatment. Data are presented as the mean ± standard deviation.

a P<0.05 compared with the CHF-SS group. SO, sham-operated; CHF, chronic heart failure; SS, sham stimulation; VNS, vagal nerve stimulation; HR, heart rate; SBP, systolic blood pressure; MBP, mean blood pressure; DBP, diastolic blood pressure; weight, body weight; LVEDV, left ventricular end-diastolic volume; LVESV, left ventricular end-systolic volume; LVFS, left ventricular fractional shortening; LVEF, left ventricular ejection fraction.
